# Investigating Priming Effects of Physical Practice on Motor Imagery-Induced Event-Related Desynchronization

**DOI:** 10.3389/fpsyg.2020.00057

**Published:** 2020-02-05

**Authors:** Mareike Daeglau, Catharina Zich, Reiner Emkes, Julius Welzel, Stefan Debener, Cornelia Kranczioch

**Affiliations:** ^1^Neuropsychology Laboratory, Department of Psychology, School of Medicine and Health Sciences, Carl von Ossietzky University of Oldenburg, Oldenburg, Germany; ^2^Neurocognition and Functional Neurorehabilitation Group, Department of Psychology, School of Medicine and Health Sciences, Carl von Ossietzky University of Oldenburg, Oldenburg, Germany; ^3^Department of Psychiatry, Oxford Centre for Human Brain Activity, Wellcome Centre for Integrative Neuroimaging, University of Oxford, Oxford, United Kingdom; ^4^Nuffield Department of Clinical Neurosciences, Wellcome Centre for Integrative Neuroimaging, Oxford Centre for Functional MRI of the Brain, University of Oxford, Oxford, United Kingdom; ^5^Department of Neurology, Christian-Albrechts-University of Kiel, Kiel, Germany; ^6^Cluster of Excellence Hearing4All, Carl von Ossietzky University of Oldenburg, Oldenburg, Germany; ^7^Research Center Neurosensory Science, Carl von Ossietzky University of Oldenburg, Oldenburg, Germany

**Keywords:** motor imagery, EEG, event-related desynchronization, physical practice, priming

## Abstract

For motor imagery (MI) to be effective, an internal representation of the to-be-imagined movement may be required. A representation can be achieved through prior motor execution (ME), but the neural correlates of MI that are primed by ME practice are currently unknown. In this study, young healthy adults performed MI practice of a unimanual visuo-motor task (Group *MI*, *n* = 19) or ME practice combined with subsequent MI practice (Group *ME&MI*, *n* = 18) while electroencephalography (EEG) was recorded. Data analysis focused on the MI-induced event-related desynchronization (ERD). Specifically, changes in the ERD and movement times (MT) between a short familiarization block of ME (Block pre-ME), conducted before the MI or the ME combined with MI practice phase, and a short block of ME conducted after the practice phase (Block post-ME) were analyzed. Neither priming effects of ME practice on MI-induced ERD were found nor performance-enhancing effects of MI practice in general. We found enhancements of the ERD and MT in Block post-ME compared to Block pre-ME, but only for Group *ME&MI*. A comparison of ME performance measures before and after the MI phase indicated however that these changes could not be attributed to the combination of ME and MI practice. The mixed results of this study may be a consequence of the considerable intra- and inter-individual differences in the ERD, introduced by specifics of the experimental setup, in particular the individual and variable task duration, and suggest that task and experimental setup can affect the interplay of ME and MI.

## Introduction

Motor imagery (MI) has been defined as a state in which a movement is solely mentally simulated (Decety, 1996). Although MI does not include any overt motor output, MI practice has been shown to affect the subsequent physical motor execution (ME) of the imagined movement, e.g., (Driskell et al., 1994; Munzert et al., 2009; Gentili et al., 2010; Wriessnegger et al., 2018). The theoretical foundation of this constitutes the neural simulation of action theory (Jeannerod, 2001). The main postulates of this theory outline that performed actions contain continuously linked covert (representational) and overt (executive) stages, whereby the covert stage includes most of the overt stage’s aspects, e.g., motor planning, to prepare the subsequent action. Moreover, the representation of an action can be consolidated via internal rehearsal without the executive part following (for a review, see O’Shea and Moran, 2017). Another key concept of this theory is the assumption that the mental representation of a specific movement and its actual execution involve cooperating (sub-)cortical networks in the brain (Jeannerod and Decety, 1995; Decety, 1996). Several neuroimaging studies have provided evidence supporting this theory (Lotze et al., 2002; Lacourse et al., 2005; Miller et al., 2010; Galdo-Alvarez et al., 2016; Mateo et al., 2018). Consequently, MI practice has been suggested to facilitate the acquisition of new motor skills, the improvement of already existing motor skills and the relearning of motor skills following a brain injury, such as stroke (e.g., Robin et al., 2007; Wei and Luo, 2010; Frank et al., 2014; Kraeutner et al., 2018). Generally, MI practice strategies can be divided into either kinesthetic or visual imagery (Neuper et al., 2005; McAvinue and Robertson, 2008). Visual MI comprises an external perspective, i.e., to imagine seeing yourself performing the given task and kinesthetic MI involves an internal perspective, i.e., to imagine the feeling that actual execution of the task produces (Annett, 1995). The activation of specific networks strongly depends on the applied MI strategy, i.e., kinesthetic but not visual MI can induce the targeted activity over sensorimotor areas (Stinear et al., 2006; Hétu et al., 2013).

Basic research aiming at systematically studying the effect of MI on ME of the same movement is generally hampered by the simplicity of tasks tailored to paretic stroke patients, such as repetitive thumb abduction (Nikulin et al., 2008; Zich et al., 2015a; Grosprêtre et al., 2016). In healthy individuals these mostly highly overlearned movements lead only to little or no measurable improvement through practice. In contrast, more complex motor tasks (Mulder et al., 2004; Golenia et al., 2014; Zabielska-Mendyk et al., 2018; Paris-Alemany et al., 2019), have the disadvantage of not being transferable to rehabilitation setups or having little everyday relevance. Finding the right balance between transferability and learnability is by far not a trivial matter. A successful step was taken by Allami et al. (2008) and their development of a novel complex visuo-motor task. Their task comprises the transport of a rectangular object with the right hand without dropping a marble loosely placed on top of it. To further modulate task difficulty the object’s orientation can be altered from trial to trial. Allami and colleagues investigated different proportions of MI and ME practice and found that high rates of MI practice preceding ME resulted in better ME behavioral performance than ME practice alone. Whether for comparable tasks ME practice in return also affects MI practice is currently unknown. Jackson et al. (2001) suggested that effective MI practice comprises some experience of the to-be-imagined movement, supposedly to have an internal representation available for recruitment during the mental simulation (Mulder et al., 2004). Based on this, we hypothesized that for the unimanual visuo-motor task ME practice shapes subsequent MI. Given the covert nature of MI, we focused in our predictions on neurophysiological correlates of ME and MI. Specifically, we tested whether a period of ME practice affects the event-related desynchronization (ERD) in the 8–30 Hz frequency range above sensorimotor areas during subsequent MI practice. The ERD reflects a power decrease of rhythmic brain activity over sensorimotor cortical areas within the mu (8–12 Hz) and beta (13–30 Hz) frequency range (Lopes da Silva and Pfurtscheller, 1999; Cheyne, 2013) and is a prominent neural correlate of ME and MI paradigms (Neuper et al., 2009; McFarland and Wolpaw, 2011; Zich et al., 2017b; Zabielska-Mendyk et al., 2018).

In this study two groups of young healthy adults underwent either a combined session of ME and MI practice (Group *ME&MI*) or a session of solely MI practice (Group *MI*), both commencing and ending with a short period of executing the practiced task. We assumed that ME practice prior to MI practice will lead to a stronger MI-induced contralateral ERD in comparison to MI practice without prior ME practice because ME practice should lead to a more pronounced internal representation of the task. In addition to this main hypothesis, given the results of Allami et al. (2008) we further hypothesized that MI practice alone will result in changes in contralateral ME-related ERD and improved task execution from pre- to post-practice. However, these changes were expected to be smaller than for the group in which ME practice preceded MI practice as a consequence of the superior internal representation of the movement acquired during ME practice.

## Methods

### Participants

A total of 42 individuals (21 women, aged 20–35 years, *M* and SD: 25.57 years ± 3.0) participated in the study. All participants reported normal or corrected-to-normal vision and were free of neurological diseases. As tested with the Edinburgh Handedness Inventory (Oldfield, 1971), all participants were right-handed. Participants were naïve to the purpose of the conducted experiments and did not have explicit knowledge about MI processes. Every participant signed an institutionally approved consent form prior to the experiment. Two datasets were discharged from analyses due to technical issues during data collection. Furthermore, three datasets were excluded due to non-compliance with task instructions, i.e., performance of ME during MI trials, as indicated by surface electromyogram (EMG, see the section “EMG Analysis” for more details). Final group sizes were 19 participants in Group *MI* (9 women, aged 20–35 years, *M* and SD: 25.89 years ± 3.9) and 18 participants in Group *ME&MI* (9 women, aged 21–29 years, *M* and SD: 25.22 years ± 1.8). The study protocol was approved by the Commission for Research Impact Assessment and Ethics of the University of Oldenburg.

### Study Design

**Figure 1:**
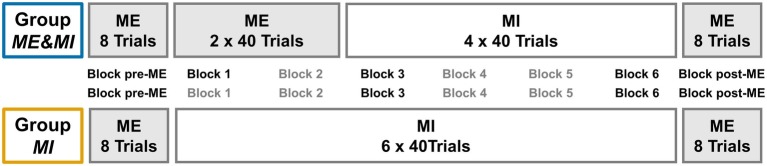
Group design. Experimental Group design. Periods of ME are indicated by a light gray background. Both groups began with eight trials of ME serving as pre-measurement (Block pre-ME). Then, Group *MI* performed six blocks of 40 trials each of MI practice (Block 1 to Block 6), while Group *ME&MI* performed two blocks of 40 trials each of ME practice (Block 1 and Block 2), followed by four blocks of 40 trials each MI practice (Block 3 to Block 6). Both groups finished with eight trials of ME (Block post-ME). Blocks subject to planned comparisons are indicated by black font.

The study design was developed to investigate differences on a within-subject as well as between-subjects basis. Therefore, participants were pseudo-randomly assigned to either of two groups. All individuals performed eight ME trials in the beginning and eight ME trials at the end of the experiment for a pre-post comparison of ME performance and ME relative ERD. In between, Group *ME&MI* performed 80 ME trials, followed by 160 MI trials, while Group *MI* performed MI for 240 trials, respectively divided into six blocks of 40 trials each (cf. Figure 1).

### Experimental Task and Set-up

**Figure 2:**
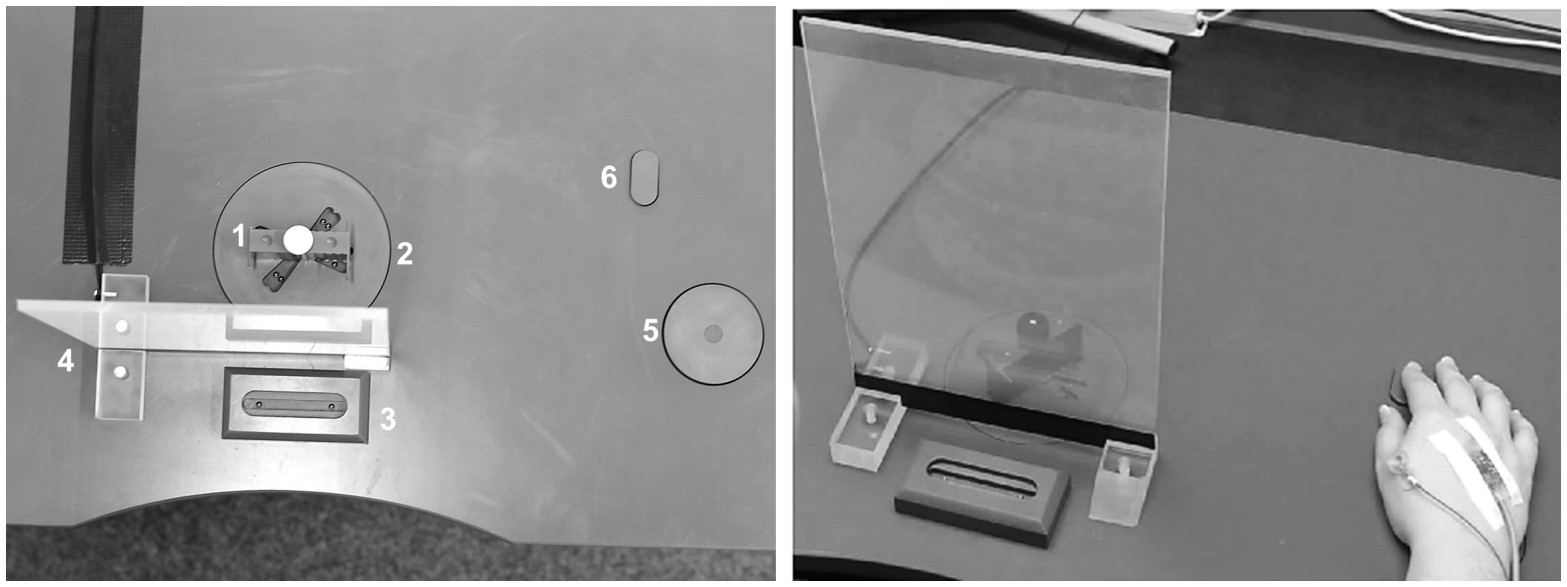
Setup. Photograph of the experimental setup. The experimental setup consisted of a customized table with two holders in the middle and two buttons on the right side (see upper photograph). For each new trial, the experimenter placed the rectangular object (1) with a marble on top in the upper, start holder (2). The start holder was equipped with slots at different angles (−22, 0, 45, and 56°) to allow for variations in the movement. Between upper (2) and lower (3) holder a glass pane (4) with an electrochromic foil was installed. Of the two buttons, the lower button (5) was a release button sending a trigger that signaled the start of the movement in motor execution (ME) trials, while pressing the upper button (6) signaled the end of the movement in both ME and motor imagery (MI) trials. A new trial would be initiated by the experimenter when object and marble were in place, and the palm of the participant’s right hand rested on the release button (right photograph). As soon as the otherwise opaque glass pane turned transparent, participants physically executed (ME) or kinesthetically imagined (MI) the movement. The movement was to reach for and grasp the object in the start holder (1 and 2), to transport it around the glass pane (4) and to place it in the lower, target holder (3), all without losing the marble. A trial was finished when the participant pressed the end button (6), which also changed the glass pane back to opaque. Then, in preparation for the next trial, the participant rested their right palm on the release-button (5), while the experimenter placed the object anew in the upper holder (2).

The task was first described by Allami et al. (2008). In short, the task comprised a unimanual two-step movement. Participants were seated in front of a table with their left hand placed comfortably on the table and their right hand resting on the table on the start position (both hands with palms down). The movement was subdivided in two basic stages: sub-movement (1) reach out and grasp the object with the right hand using a precision grip (thumb and index finger), sub-movement (2) transport the object to its target location. Participants were given written and verbal instructions, the latter in form of a video, demonstrating the task from the first-person perspective. For ME trials participants were instructed to perform the two-step movement as fast and accurate as possible. Crucially, this led to intra- and inter-individual differences in trial durations, i.e., timings are inherently different across trials. For MI trials participants were instructed to kinesthetically imagine the same movement in a pace comparable to the pace during ME. To emphasize the difference between visual and kinesthetic MI the short version of the kinesthetic and visual imagery questionnaire (KVIQ; Malouin et al., 2007) was conducted before the experiment. To increase the task difficulty a marble was placed on a low indentation upon the object. Across trials the start orientation of the object (−22, 0, 45 and 56°) was altered pseudo-randomly. The orientation of the target location (0°) was constant. To avoid a priori knowledge about the orientation of the object before the beginning of the trial, a glass pane (height: 30.5 cm; width: 20.0 cm; depth: 0.5 cm) with an electrochromic foil (MagicFoil, MediaVision, Wilnsdorf, Germany) attached was installed on the table in between the participant and the start slot of the grasping object. Between trials (pseudo-randomized inter-trial-interval range of 4–4.75 s in 0.25 s steps) the glass pane was opaque, allowing the experimenter to place the object in its starting position before each trial, while hiding the object’s position from the participant (cf. Figure 2). With the beginning of the trial the state of the glass pane changed to transparent, serving as signal for the participants to immediately start to execute or imagine the movement. Both, ME and MI trials ended when the participant pressed a button embedded in the table with their right index finger. With the button press, the glass pane became opaque again. For temporally tracking the individual movement stages the table was equipped with additional trigger mechanisms. ME trials contained the triggers start of trial (glass pane transparent), start of movement (moving the right hand away from the starting position), end of sub-movement 1 (lifting the object of the table), end of sub-movement 2 (placing the object in the target position), and end of trial (button press). MI trials contained the triggers start of trial (glass pane transparent) and end of trial (button press). All triggers were controlled with an Arduino Nano microcontroller board and Presentation software (Version 17.0, Neurobehavioral Systems, Inc., Berkeley, CA, USA, RRID:SCR_002521) via a serial port connection.

Participants were instructed to perform the task as fast as possible but without losing the marble. This resulted in a large range of individual’s trial durations and therefore also in differences in the duration of the whole experiment. The mean duration of the experimental task obtained from all 256 trials per subject across both groups was 30 min ± 1, ranging from 20 to 55 min. The mean experiment duration for Group *MI* was 32 min ± 2 (range 20–55 min) and for Group *ME&MI* 27 min ± 1 (range 20–35). Participants were asked to rate their level of motivation and tiredness before and after the experiment as well as between blocks on visual analogue scales (not motivated/tired at all – very motivated/tired). After the experiment participants assessed their MI experience regarding easiness (very easy – easy – neutral – difficult – very difficult) and vividness (as vivid as when executing – almost as vivid as when executing – reasonably vivid – moderately vivid – no sensation) on Likert scales, that also allowed for ratings in-between scale intervals.

### Data Acquisition

Electroencephalography (EEG) data were acquired from 64 Ag/AgCl electrodes using an equidistant infracerebral electrode layout with a central frontopolar site as ground and a nose tip reference (Easycap, Herrsching, Germany). Bipolar surface EMG was simultaneously recorded from both hands and arms by placing Ag/AgCl electrodes over the muscle belly and the proximal base of the *m*. *flexor digitorum superficialis* and *m*. *adductor pollicis* with the reference on the left collarbone. Both EEG and EMG data were recorded using a BrainAmp amplifier system (BrainProducts, Gilching, Germany). Data were obtained with an amplitude resolution of 0.1 μV and a sampling rate of 500 Hz with online analogue filter settings of 0.016–250 Hz. Electrode impedances were maintained below 10 kΩ for the EEG and below 100 kΩ for the EMG before data acquisition. Lab streaming layer software (UCSD Swartz Center for Computational Neuroscience, 2017) was used to synchronize the EEG and EMG data as well as the experimental events. In addition to that, but with no results reported here, an inertial measurement unit (IMU) device (Adafruit BNO055 Absolute Orientation Sensor, Adafruit Industries, New York City, New York) was attached to the participants right back of the hand and electrodermal activity (EDA) was recorded during the experiment from the left index and middle finger and resting state EEG lasting 2 min. was recoded after the experiment.

### Behavioral Analysis

Movement time (MT_total_; i.e., time from start of trial to end of trial) was calculated for each trial. To account for the multi-stage character of the movement, trial durations for ME trials were further divided into the following sub-movement times: movement time 1 (MT1; i.e., time from lifting the hand until lifting the object) and movement time 2 (MT2; i.e., time from lifting the object until placing it in its target position). MI trials containing actual movements as measured with EMG (see the section “EMG Analysis”) and ME trials in which the marble was dropped (mean marble drops 1.97 ± 0.15, range 0–4 in 16 trials) were excluded from behavioral and EEG data analyses.

### Electromyogram Analysis

EMG data were filtered with a cut-off frequency of 25 Hz using a high-pass finite-impulse response filter with a hamming window (filter order: 264). Noise removal was performed via wavelet denoising (wavelet signal denoiser toolbox, MathWorks, Natick, MA, USA) with a Daubechies 4 (dB4) wavelet. EMG data were then segmented from −3 s relative to the start of each trial to the end of the trial. For each trial the standard deviation and the 250-samples centered moving standard deviation were calculated. Trials in which the moving standard deviation exceeded the standard deviation of the trial by the factor 1.5 at any point were considered to contain movement artifacts and excluded from further analyses (See section “Results” for more details).

### EEG Analysis

EEG data were preprocessed with the EEGLAB toolbox Version 14.1.1 (Delorme and Makeig, 2004) for MATLAB (Version 9.3; MathWorks, Natick, MA, USA, RRID:SCR_001622). Artifact correction was performed using independent components analysis (ICA). A copy of the EEG data was first high-pass filtered (1 Hz, FIR, hamming window, filter order: 414), down-sampled to 250 Hz and low-pass filtered (40 Hz, FIR, hamming window, filter order: 166) and segmented into consecutive one-second epochs. Segments containing artifacts were removed (EEGLAB functions pop_jointprob.m, pop_rejkurt.m, both SD = 3). Remaining data were submitted to the extended infomax algorithm to estimate the unmixing weights of 64 independent components. The unmixing matrix obtained from this procedure was applied to the original unfiltered EEG dataset for selection and rejection of components representing stereotypical artifacts. Components reflecting eye blinks and lateral eye movements were identified using the fully-automated Eye-Catch approach (Bigdely-Shamlo et al., 2013). Additionally, components reflecting cardiac activity were identified by visual inspection and removed accordingly. Artifact corrected EEG data were low-pass filtered with a finite-impulse response filter and a cut-off frequency of 30 Hz (hamming window, filter order 220, Fs = 500 Hz), down sampled, and subsequently high-pass filtered with a finite-impulse response filter and a cut-off frequency of 8 Hz (hamming window, filter order 414, Fs = 250 Hz). The filtered data were down-sampled to 100 Hz to reduce computational demand. Identification of improbable channels was conducted using the EEGLAB extension *trimOutlier*^1^ with an upper and lower boundary of two standard deviations of the mean standard deviation across all channels (mean channels identified: 3.03 ± 0.21, range 1–5 channels). After the data were re-referenced to common average, bad channel signals were replaced by spherical interpolation. Then data were segmented from –3 s relative to the start of each trial to the maximal trial length across all trials of each participant. Artefactual epochs as indicated by the joint probability within each of the experimental blocks (EEGLAB function pop_jointprob.m, pop_rejkurt.m, both SD = 3) and epochs flagged by the EMG analysis were discarded from further analyses. Subsequently, the artifact trimmed EEG data was epoched into trials, with epoch length determined by the specific trial duration.

**Figure 3:**
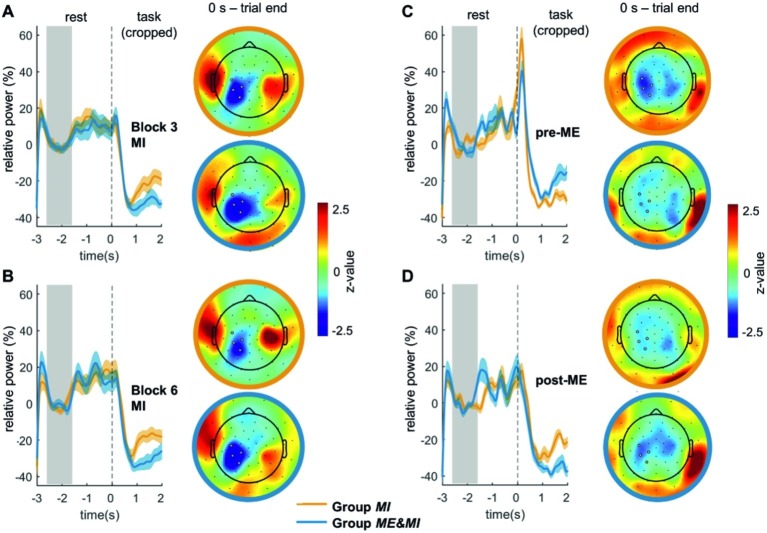
ERD time courses and mean topographies for ME and MI. Illustrated are the mean relative ERD per group and standard error for **(A)** Block pre-ME, **(B)** Block post-ME, **(C)** Block 3 (corresponding to first MI block for Group *ME&MI* and to third MI block for Group *MI*), **(D)** Block 6 (corresponding to fourth MI block for Group ME&MI and to sixth MI block for Group MI). As trial length varied considerably, the ERD traces comprise only the first 2 s of the task phase that were available for all trials. The gray area indicates the baseline period, and the dashed black line represents the start of the trial. Topographies show the distribution of relative ERD averaged across trials and participants. Topographies are based on individual single trial durations as described in the methods. Topographies were z-transformed within each condition for illustration purposes. Note that positive *z*-values can result from the z-transformation; they correspond however to negative relative power values. White dots highlight the electrodes of the contralateral ROI from which the mean relative ERDs were derived for statistical analysis.

The task-related event-related desynchronization (ERD) was extracted following the procedure proposed by Lopes da Silva and Pfurtscheller (1999). Due to the variability in trial duration, baseline normalization was performed on single trial level instead of the averaged signal. The baseline was defined as −2.6 to −1.6 s before the start of each trial. Smoothing was conducted using a Gaussian window of width 40 that was convolved with the EEG signal. The region of interest (ROI) was defined based on previous studies (Zich et al., 2015a,b), and included posterior frontal, central, and anterior parietal areas of the left hemisphere, thus covering sensorimotor areas (see Figure 3). The resulting signal was averaged over those channels. Additionally, epochs containing values exceeding the median power over all trials per subject by three standard deviations were removed from the data (9.95 trials ± 1.17 affected, range: 0–33 trials). Then, for each trial, all data points below the trials’ baseline value were averaged. This procedure was adopted to account for the considerable variability in single trial durations and for fluctuations in the trial ERD time course. Finally, data were averaged block-wise over trials. The resulting final feature will be referred to as relative ERD. The block-wise averages included all valid trials of the corresponding block, with up to eight trials for pre and post and up to 40 trials for Block 1 – Block 6 (cf. Figure 1). On average, a total of 213.5 trials ± 1.6 per subject (range 195–232 trials) were used for analysis.

### Statistical Analyses

#### Group Characteristics

Both groups were compared regarding motivation and tiredness prior to the experiment, as well as KVIQ scores, MI easiness and MI vividness ratings by means of Bayesian independent samples *t*-tests.

#### Motor Imagery

A (2 × 1)-mixed ANCOVA with group (Group *MI*, Group *ME&MI*) as between-subject factor, block (Group *MI* Block 1, Group *ME&MI* Block 3) as within-subject factor and KVIQ scores as covariate was performed comparing the first MI blocks between groups to test the potential impact of ME on the subsequent MI-induced relative ERD. A (2 × 2)-mixed ANCOVA with group (Group *MI*, Group *ME&MI*) as between-subject factor, block (Block 3, Block 6) as within-subject factor and KVIQ scores as covariate was conducted for the relative ERD to test for the presence of a gain in relative ERD from Block 3 to Block 6 induced by MI practice. To ensure a fair comparison, the relative ERD of Block 3 was compared between groups by means of a Bayesian independent samples *t*-test prior to the ANCOVA.

#### Motor Execution

Relative ERD and MT for both groups calculated from up to eight ME trials in the beginning of the experiment (Block pre-ME) were first compared by means of Bayesian independent samples *t*-tests to ensure that Block pre-ME values were comparable between groups. Then two separate (2 × 2)-mixed ANOVAs with group (Group *MI*, Group *ME&MI*) as between-subject factor and block (Block pre-ME, Block post-ME) as within-subject factor were implemented to test for differences in relative ERD and MT_total_. In addition, for MT1 and MT2, two separate (2 × 2)-mixed ANOVAs with group (Group *MI*, Group *ME&MI*) as between-subject factor and block (Block pre-ME, Block post-ME) as within-subject factors were implemented.

For Group *ME&MI* the relative ERDs and MTs obtained from Block pre-ME and Block post-ME were compared to the last ME block before the MI phase (i.e., second ME practice block, Block 2) by means of paired samples *t*-tests to explore the progress of ERD changes over time. To ensure a fair comparison, for Block 2, the number of trials entering this analysis was matched to the individual number of valid trials of Block post-ME. Trials were randomly selected from all valid trials of Block 2.

To test whether changes between Block pre-ME and Block post-ME (i.e., post minus pre and denoted as Δ) at neural and behavioral levels are related, across-group Spearman correlations were conducted for pre-post changes of relative ERD and pre-post changes of either MT_total_, MT1, or MT2.

For all analyses, in case that sphericity was violated Greenhouse-Geisser-correction was applied as implemented in the R-package ez (version 4.4-0). *Post hoc* comparisons were conducted using two-tailed (paired) *t*-tests except where otherwise stated. Multiple pairwise comparisons were corrected for by the Holm-Bonferroni method according to the number of performed tests (Holm, 1979). All numerical values are reported as mean ± SE. Effect sizes are reported as Eta-squared (η^2^) for ANOVAs and Cohen’s d (*d*) for *t*-tests. All Frequentist statistics were conducted as implemented in RStudio (Version 1.1.463; R Core Team, 2017, RRID:SCR_001905). All Bayesian statistics were performed with the free software JASP using default priors (Version 0.9.2.0; JASP Team, 2019, RRID:SCR_015823).

## Results

### Group Characteristics

Both groups were compared regarding motivation and tiredness prior to the experiment, as well as MI easiness, MI vividness ratings and KVIQ scores (cf. Table 1). Bayesian independent samples *t*-tests suggested no difference between both groups regarding initial motivation (BF_10_ = 0.36), tiredness (BF_10_ = 0.40), MI easiness (BF_10_ = 0.38) and MI vividness ratings (BF_10_ = 0.33). Regarding KVIQ scores there was anecdotal evidence for a difference between both groups (BF_10_ = 1.42), consequently KVIQ scores were included as covariate in comparisons of the MI-induced relative ERD.

**Table 1:** Group characteristics for self-reported motivation, tiredness, MI easiness, MI vividness, and KVIQ scores (*M* ± SD).

	Self-reported group characteristics				
	Motivation	Tiredness	MI easiness	MI vividness	KVIQ scores
Group *MI*	81.1% ± 10.4	26.7% ± 21.9	2.9 ± 0.6	3.1 ± 0.7	33.3 ± 4.6
Group *MI&ME*	83.5% ± 14.5	21.9% ± 16.3	3.1 ± 0.8	3.1 ± 0.7	35.9 ± 3.0

### Electromyogram Activity

EMG activity was analyzed to identify movements during the baseline period of all trials and the MI period of the MI trials. Three participants were identified as outliers (data points outside 1.5 times the interquartile range above the upper quartile) with more than 50 trials being marked as movement in the MI period (range 51–68 trials). These individuals were excluded from further analyses. For the remaining participants on average 6.92 trials ± 1.05 were identified to contain movement (range 0–24 trials). Those trials were excluded from further analyses.

### EEG

EEG analyses are based on MI induced relative ERD (Group *MI*: Block 1, Block 3, Block 6; Group *ME&MI*: Block 3, Block 6) and ME relative ERD (Group *MI*: pre-ME, Block post-ME; Group *ME&MI*: Block pre-ME, Block 2, Block post-ME).

#### Motor Imagery

**Figure 4:**
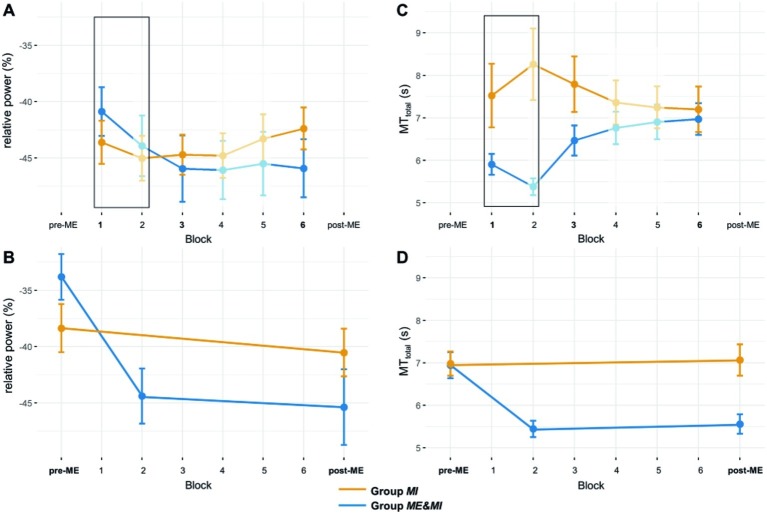
ERD and MT_total_ for all experimental blocks. **(A)** Mean ERD and standard error per group for Block 1 to Block 6. The black rectangle marks blocks in which groups performed different tasks, i.e., Group *ME&MI* performed ME, and Group *MI* performed MI. Blocks without statistical comparisons are displayed transparent. **(B)** Mean ERD and standard error per group for Block pre-ME and Block post-ME. For Group *ME&MI* the mean ERD derived from a subset of trials of the second ME block (Block 2) is also shown (see section “Methods” for details). **(C)** Mean movement time and standard error per group for Block 1 to Block 6. The black rectangle marks blocks in which groups performed different tasks, i.e., Group *ME&MI* performed ME, and Group *MI* performed MI. Blocks without statistical comparisons are displayed in transparent. **(D)** Mean movement time and standard error per group for Block pre-ME and Block post-ME. For Group *ME&MI*, the MT_total_ time derived from a subset of trials of the second ME block (Block 2) is also shown (see section “Methods” for details).

Motor imagery was associated with ERD centered over centro-parietal electrodes. Time courses of Block 3 and Block 6 and corresponding topographies are shown in Figures 3A,B. MI-induced relative ERDs for the two groups are shown in Figure 4A.

##### Impact of ME Practice on Subsequent MI-induced Relative ERD

To investigate the potential impact of ME on the subsequent MI-induced relative ERD, the first MI block of each group (Group MI: Block 1, Group ME&MI: Block 3) were compared. The statistical comparison, i.e., (2 × 1) mixed ANCOVA, indicated no significant difference in ERDs between groups after controlling for KVIQ scores (*F*_1,35_ = 0.47, *p* = 0.50, *η*^2^ = 0.01).

##### Practice Effects From Block 3 to Block 6 for MI-induced Relative ERD

To ensure a fair analysis of any change in relative ERD induced by MI practice, the relative ERD of Block 3 of the two groups was compared first. The Bayesian independent samples *t*-test suggested anecdotal evidence in favor of H0 (BF_10_ = 0.34), that is, no difference between both groups for Block 3 in MI-induced ERD. Following, a (2 × 2)-mixed ANCOVA with group (Group *MI*, Group *ME&MI*) as between-subject factor, block (Block 3, Block 6) as within-subject factor, KVIQ scores as covariate and relative ERD as dependent variable was conducted. No significant main effect or interaction could be observed (cf. Table 2; Figure 4A).

**Table 2:** 2 × 2 ANCOVA for MI-induced ERD (Block 3, Block 6) and KVIQ scores as covariate.

	MI-induced ERD			
	df	*F*	*p*	*η*^2^
Group	1,35	0.63	0.43	0.02
Block	1,35	3.53	0.07	0.004
Group × Block	1,35	3.18	0.08	0.003

#### Motor Execution

Motor execution was associated with an ERD centered over centro-parietal electrodes. Time courses of Block pre-ME and Block post-ME and corresponding topographies are shown in Figures 3C,D. Relative ERDs of Block pre-ME and Block post-ME and, for Group *ME&MI*, additionally of Block 2, are shown in Figure 4B.

##### Practice Effects From Block pre-ME to Block post-ME for Relative ERD

A Bayesian independent samples *t*-test suggested anecdotal evidence in favor of H0 (BF_10_ = 0.796), that is, no initial difference between both groups in relative ERD for Block pre-ME. The (2 × 2)-mixed ANOVA with group (Group *MI*, Group *ME&MI*) as between-subject factor and block (Block pre-ME, Block post-ME) as within-subject factor and relative ERD as dependent variable indicated a main effect of block (*F*_1,35_ = 11.57, *p* = 0.002, *η*^2^ = 0.097). Moreover, a significant block-by-group interaction (*F*_1,35_ = 5.62, *p* = 0.023, *η*^2^ = 0.0497) was observed (cf. Table 3; Figure 4B). To further investigate the interaction, the data was split by group and two paired *t*-tests were conducted. Results indicated a significant difference between Block pre-ME and Block post-ME for Group *ME&MI* (pre-ME: −33.80% ± 2.03, post-ME: −45.38% ± 3.36, *t*_17_ = 3.29, *p* = 0.004, *d* = 0.77), but not for Group *MI* (pre-ME: −38.36% ± 2.15, post-ME: −40.53% ± 2.12, *t*_18_ = 1.12, *p* = 0.28, *d* = 0.26).

**Table 3:** 2 × 2 ANOVA for ME ERD (Block pre-ME, Block post-ME).

	ME ERD			
	df	*F*	*p*	*η*^2^
Group	1,35	0.003	0.96	5.1e^−05^
Block	1,35	11.58	**0.002**	0.10
Group × Block	1,35	5.62	**0.023**	0.05

*p ≤ 0.05 are highlighted in boldface*.

These results demonstrate significantly stronger relative ERD in Block post-ME compared to Block pre-ME for Group *ME&MI* (cf. Figure 4B).

##### Origin of Practice Effects for ME Relative ERD

We further explored the nature of the observed significant enhancement in relative ERD between Block pre-ME and Block post-ME for Group *ME&MI*. We specifically asked whether the enhancement resulted from ME practice, MI practice or the combined effects of ME and MI practice. To answer this question, Block pre-ME and Block post-ME relative ERDs were compared to the relative ERD derived from the second ME practice block (i.e., Block 2). The paired samples *t*-test between Block pre-ME and Block 2 was found to be significant (pre-ME: −33.80% ± 2.03, block two: −43.94% ± 2.70, *t*_16_ = 4.14, *p* = 0.002, *d* = 0.98). The paired samples *t*-test between Block 2 and Block post-ME was not significant (Block 2: −43.94% ± 2.70, Block post-ME: −45.38% ± 3.36, *t*_16_ = 0.69, *p* = 0.93, *d* = 0.16).

These results indicate that the significant enhancement in relative ERD between Block pre-ME and post-ME for Group *ME&MI* is driven by ME practice (cf. Figure 4B).

### Behavioral Performance

Analysis of behavioral data focused on total movement time (MT_total_; i.e., time from start of trial to end of trial; equivalent to trial duration), MT1 (i.e., time from lifting the hand until lifting the object) and MT2 (i.e., time from lifting the object until placing it in its target position). For Group *MI* this comparison included Block pre-ME and Block post-ME, for Group *ME&MI* Block pre-ME, Block 2 and Block post-ME.

#### MT_total_

Mean movement times ranged between approximately 5.5 and 8.5 s. A Bayesian independent samples *t*-test was run to test for initial differences between groups. Results suggested moderate evidence in favor of H0 (BF_10_ = 0.32), that is, no initial difference between both groups for Block pre-ME in MT_total_. Group means of MT_total_ for pre-ME, post ME and for Block 1 to Block 6 are displayed in Figures 4C,D.

##### Practice Effects From Block pre-ME to Block post-ME for MT_total_

The (2 × 2)-mixed ANOVA with group as between-subject factor, block (Block pre-ME, Block post-ME) as within-subject factor and MT_total_ as dependent variable indicated a significant main effect of block (*F*_1,35_ = 11.78, *p* = 0.002, *η*^2^ = 0.06) and a significant block-by-group interaction (*F*_1,35_ = 15.92, *p* < 0.001, *η*^2^ = 0.08) (see Table 4). To further investigate the interaction-effect the data was split by group and two paired *t*-tests were performed. These analyses revealed a significant pre-post effect for Group *ME&MI* (Block pre-ME: 6.94 s ± 0.31, Block post-ME: 5.56 s ± 0.23, *t*_17_ = 4.82, *p* < 0.001, *d* = 1.14), but not for Group *MI* (Block pre-ME: 6.98 s ± 0.28, Block post-ME: 7.07 s ± 0.37, *t*_18_ = −0.36, *p* = 0.726, *d* = −0.08).

**Table 4:** 2 × 2 ANOVAs for MT_total_, MT1, and MT2 (Block pre-ME, Block post-ME).

		MT_total_			MT1			MT2		
	df	*F*	*p*	*η*^2^	*F*	*p*	*η*^2^	*F*	*p*	*η*^2^
Group	1,35	4.02	0.053	0.09	1.66	0.21	0.03	4.01	0.053	0.09
Block	1,35	11.78	**0.002**	0.06	0.95	0.34	0.01	8.19	**0.007**	0.04
Group × block	1,35	15.92	**<0.001**	0.08	10.15	**0.003**	0.07	15.65	**<0.001**	0.07

*p ≤ 0.05 are highlighted in boldface*.

The results confirm shorter MT_total_ durations in Block post-ME in comparison to Block pre-ME for Group *ME&MI* only (cf. Figure 4D).

##### Origin of Practice Effects for MT_total_

We explored whether the significant decrease in MT_total_ between Block pre-ME and Block post-ME for Group *ME&MI* was already apparent before MI practice. To this end, Block pre-ME and Block post-ME MT_total_ was compared to the MT_total_ derived from a subset of trials of the second ME practice block (Block 2, see above for details). The paired samples *t*-test between Block pre-ME and Block 2 was significant (Block pre-ME: 6.94 s ± 0.31, Block 2: 5.38 s ± 0.20, *t*_16_ = 5.42, *p* < 0.001, *d* = 1.28). The paired samples *t*-test between Block 2 and Block post-ME was not significant (Block 2: 5.38 s ± 0.20, Block post-ME: 5.56 s ± 0.23, *t*_16_ = 1.09, *p* = 0.58, *d* = 0.26).

These results indicate that the significant enhancement in MT_total_ between Block pre-ME and Block post-ME for Group *ME&MI* is driven by ME practice (cf. Figure 4D).

#### Sub-Divided Movement Times

To explore whether the observed behavioral effects are specific to a particular movement stage, we further compared movement time 1 and 2 (MT1 and MT2).

##### Practice Effects From Block pre-ME to Block post-ME for MT1 and MT2

A Bayesian independent samples *t*-tests was run to test for initial differences between groups. Results suggested no initial difference between groups for Block pre-ME in MT1 and MT2 (MT1: BF_10_ = 0.35; MT2: BF_10_ = 0.33). To investigate both MT1 and MT2 across time and groups, two (2 × 2)-mixed ANOVAs with group (Group *MI*, Group *ME&MI*) as between-subject factor and block (Block pre-ME, Block post-ME) as within-subject factors were conducted.

For MT1 the group-by-block interaction was significant (*F*_1,35_ = 10.15, *p* = 0.003, *η*^2^ = 0.07, Table 4). To further investigate this effect, the data were split by group and two paired *t*-tests were performed resulting in a significant effect for the Group *ME&MI* (Block pre-ME: 1.37 s ± 0.07, Block post-ME: 1.15 s ± 0.06, *t*_17_ = 3.10, *p* = 0.012, *d* = 0.73), but not for the Group *MI* (Block pre-ME: 1.32 s ± 0.07, Block post-ME: 1.43 s ± 0.08, *t*_18_ = −1.46, *p* = 0.32, *d* = −0.34).

For MT2 the main effect block was significant (*F*_1,35_ = 8.19, *p* = 0.007, *η*^2^ = 0.04). In addition, the block-by-group interaction was significant (*F*_1,35_ = 15.65, *p* < 0.01, *η*^2^ = 0.07, Table 4). To further investigate the interaction, the data were split by group and two paired *t*-tests were performed resulting in a significant effect for the Group *ME&MI* (Block pre-ME: 3.35 s ± 0.18, Block post-ME: 2.72 s ± 0.12, *t*_17_ = 4.26, *p* < 0.001, *d* = 1.0), but not for Group *MI* (Block pre-ME: 3.39 s ± 0.14, Block post-ME: 3.48 s ± 0.17, *t*_18_ = −0.83, *p* = 0.84, *d* = −0.19).

Results indicate that similar to MT_total_, both MT1 and MT2 were shorter in Block post-ME than in Block pre-ME for Group *ME&MI*, but not for Group *MI*.

##### Origin of Practice Effects on MT1 and MT2

We further explored whether the significant decrease in MT1 and MT2 between Block pre-ME and Block post-ME for Group *ME&MI* was already apparent before MI practice. Therefore, Block pre-ME and Block post-ME values were compared to the values derived from a subset of trials of the second ME practice block (Block 2, see above for details).

The paired samples *t*-test for MT1 between Block pre-ME and Block 2 was significant (Block pre-ME: 1.37 s ± 0.07, Block 2: 0.97 s ± 0.03, *t*_16_ = 5.10, *p* < 0.001, *d* = 1.20). The paired samples *t*-test between Block 2 and Block post-ME was also significant (Block 2: 0.97 s ± 0.03, Block post-ME: 1.15 s ± 0.06, *t*_16_ = −2.94, *p* = 0.02, *d* = −0.69). Note that from Block 2 to Block post-ME participants became significantly slower for MT1.

For MT2, the paired samples *t*-test between Block pre-ME and Block 2 was significant (Block pre-ME: 3.35 s ± 0.18, Block 2: 2.83 s ± 0.13, *t*_16_ = 3.07, *p* = 0.01, *d* = 0.72), while it was not significant between Block 2 and Block post-ME (Block 2: 2.83 s ± 0.13, Block post-ME: 2.72 s ± 0.12, *t*_16_ = 1.09, *p* = 0.58, *d* = 0.26).

Together these results indicate that similar to MT_total_, for MT1 and MT2 the gain in behavior between Block pre-ME and Block post-ME for Group *ME&MI* is driven by ME practice. Interestingly, while for MT2, similar to MT_total,_ no change was evident from Block 2 to post-ME, the pattern was different for MT1. Specific to MT1, movement duration increased significantly from Block 2 to post-ME. The increase was however considerably smaller than the decrease from pre-ME to Block 2, resulting in the net decrease seen from pre-ME to post-ME MT1.

### Relationship Between Relative ERD and Behavioral Data

Across-group Spearman correlations were conducted for pre-post changes of relative ERD and pre-post changes of either MT_total_, MT1, or MT2 to test for brain-behavior relationships. No significant associations were obtained between ΔERD and ΔMT_total_ (ρ_37_ = −0.09, *p* > 0.9), ΔERD and ΔMT1 (ρ_37_ = −0.003, *p* > 0.9), and ΔERD and ΔMT2 (ρ_37_ = 0.04, *p* > 0.9).

## Discussion

In the present study, we examined how ME practice of a unilateral hand movement affects the subsequent MI-induced relative ERD in the contralateral sensorimotor cortex. Further, we investigated ME-induced relative ERD and related behavioral measures, i.e., movement durations of the same movement in a pre-post comparison.

Our predictions were threefold: H1. The MI-induced relative ERD is stronger for the group with prior ME practice (Group *ME&MI*) than for the group without prior ME practice (Group *MI*) in MI Block 3 and MI Block 6. This was addressed by comparing the relative ERDs of the first MI blocks (Group *ME&MI*: Block 3, Group *MI*: Block 1) and the last MI blocks across groups (Block 6) H2. The ME-induced relative ERD in Block post-ME is stronger than in Block pre-ME for both groups, whereby this effect is more pronounced in Group *ME&MI* than in Group *MI*. This hypothesis was investigated by comparing the relative ERDs of Block pre-ME and Block post-ME across both groups; and H3. Movement durations are shorter in Block post-ME than in Block pre-ME for both groups, whereby this effect is more pronounced in Group *ME&MI* than in Group *MI*. Therefore, we compared the total movement (MT_total_) and sub-movement durations (MT1, MT2) between Block pre-ME and Block post-ME.

Inconsistent with H1, we did not find a significant difference in the MI-induced relative ERD between the groups, neither in the initial MI block nor in the last MI block. For H2 and H3, we found significant group-by-block interactions for both, the ME-induced relative ERD and movement times, indicating stronger relative ERDs and shorter trial durations from Block pre-ME to Block post-ME, but only for Group *ME&MI*. However, these changes were already present before the extensive MI-phase, and thus, no evidence was found for a contribution of MI practice to these changes.

### Priming Effects of Motor Execution on Motor Imagery

We did not find evidence that prior ME practice leads to a stronger subsequent MI-induced relative ERD of the very same movement in comparison to no prior ME practice. This result is unexpected given the report of priming effects of MI on subsequent ME on the neural level (Pascual-Leone et al., 1995; Allami et al., 2014) even though these studies focused on the opposite direction and on the event-related potential (ERP) rather than the ERD. Moreover, this is contrary to two studies conducted by Wriessnegger and colleagues, who suggested that sports exercises potentially lead to a boost in subsequent MI patterns (Wriessnegger et al., 2014) including the ERD (Wriessnegger et al., 2018) and reports of behavioral priming effects of ME on MI, such as the ease of MI (Williams et al., 2011). In the following we will discuss possible explanations for this outcome.

First, it cannot be ruled out that the groups differed in their initial MI-induced relative ERD, and that the ME practice performed in one of the groups equalized this difference. The study’s design did not allow for an empirical test of this possibility. However, comparisons of the initial ME-induced relative ERDs and respective movement times showed no significant differences between both groups, making it an unlikely explanation.

Second, it may be the case that the eight-trial Block pre-ME run in Group *MI* to familiarize with the task was sufficient to form a good internal representation for mental simulation, or that the task allowed to extrapolate a good internal representation from everyday experience (Mulder et al., 2004). In both cases the benefit of additional ME practice for MI would be minimized. The results of Allami and colleagues are in line with the possibility that a sufficiently good mental representation of the task can be acquired without extensive practice. In their study, five familiarization ME trials prior to the MI practice phase sufficed to make MI practice accurate enough to affect motor-related ERPs and to enhance ME performance measures (Allami et al., 2014). Yet similar to the present study, it remains open whether forming the mental representation required familiarization trials at all. Moreover, we cannot rule out the possibility that watching the instruction video primed task performance in both groups, although this scenario is rather unlikely given the videos limited duration (see e.g. Buccino, 2014; Polzien et al., 2019).

Another aspect may be the structure of the experiment, combining blocks of ME with consecutive blocks of MI practice. This design was chosen because it would also be suitable for a neurofeedback setup with an extensive ME phase prior to MI neurofeedback (NF) practice. Yet results from a behavioral study indicate that a contingent combination of ME and MI practice in an alternating design may yield better effects (McBride and Rothstein, 1979). It was found that MI practice alone was not as effective as ME practice and that ME practice alone was not as effective as combined interleaved MI and ME practice. Whether for the present setup an alternating design would boost the MI-induced ERD and would thus be promising for MI NF designs should be addressed in future studies.

### Combination of Motor Execution and Motor Imagery to Boost Motor Execution Performance

Consistent with H2 and H3, we found significant group-by-block interactions for both, the ME-induced relative ERD and movement durations (MT_total_, MT1, MT2). However, further investigation of the interactions revealed that significant changes were restricted to Group *ME&MI*. Moreover, although relative ERD was significantly stronger and movement durations were significantly shorter in Block post-ME compared to Block pre-ME in this group, these changes were already apparent before MI practice. Thus, they cannot be related to the phase of MI practice subsequent to the ME practice phase. This contrasts with several studies in the neuro-rehabilitation context combining MI practice with ME practice, and finding that the combination improves subsequent ME performance in comparison to MI practice only (Malouin et al., 2013; Bajaj et al., 2015), but because of the between-subject design and the possibility of initial group differences in these latter studies, MI and ME influences cannot be separated.

Another possible explanation for the discrepancy between studies suggesting a beneficial effect of combining ME and MI and our study may be that we decided against a temporally defined, movement specific pre-trial preparation phase. This is in contrast to the setup of Allami et al. (2008, 2014) that formed the basis of the present studies’ setup but made use of an S1/S2 structure. Participants were instructed to keep their eyes closed before each trial. With a first tone (S1) participants opened their eyes, another sound (S2) indicated the start of the actual trial. As participants could see the to-be-transported block and its orientation between S1 and S2, they had the opportunity to specifically prepare the subsequent physically or mentally performed action. In the present setup, in order to prevent orientation-specific preparation and the premature occurrence of the associated ERD, the task started as soon as the glass pane turned transparent revealing the orientation of the object. The absence of an explicit pre-trial preparation may have a negative impact on MI performance and thus practice effects, while having no or little effects on ME practice effects. This idea could be tested in a direct comparison of an S1/S2 setup and a setup without S1/S2 phase.

Interestingly, for Group *ME&MI*, MT1 declined from Block 2 (i.e., the last ME block prior to the MI-phase) to Block post-ME. That is, MT1 became longer after MI-practice. In contrast, MT2 did not change significantly after the MI-phase. If anything, on a descriptive level a slight decrease was evident. During MI practice, participants may have focused more on the second, more challenging part of the motion sequence (i.e., transporting the device to its target position), while neglecting the easier reaching part. This interpretation is at least partially supported by Allami et al. (2008) who found enhancements in both movement times, but a stronger effect for MT2. The opposite direction of change observed for MT1 in the present study, and potentially also the reduction in the MT2 effect, might relate to the absence of the pre-trial preparation phase as discussed above. Another possible reason might be that because of differences in the studies’ aims and design, the way in which behavioral measurements were extracted differs profoundly between the present study and Allami et al. (2008). In the present study, comparisons were based on mean movement times from four to eight (*M*: 6.6 trials) trials for Block pre-ME and Block post-ME. The results of Allami et al. (2008) are based on one value per measurement point, derived from a fitting equation that resulted from an exponential Logreg function. Moreover, while in the present study it was important to capture performance prior to an extensive phase of task practice - as implemented with Block pre-ME – the focus of Allami et al. (2008) was to capture ME performance following different numbers of MI practice trials. Hence, in contrast to the present study, their first reported measurement point does not correspond to the beginning of the ME practice phase, but to a timepoint following at least 60 trials of MI practice. Taken together, at present it is not possible to draw firm conclusions regarding the origin of discrepancies in findings between the present study and related work. The discrepancies however emphasize that for the visuo-motor task tested here even supposedly small changes in the setup of task or experiment might have a large impact on the outcome.

### Replacement of Physical Practice Through Motor Imagery for Learning a Visuomotor Task

We did not find a significant difference between MI practice Block 3 and Block 6 of the MI-induced relative ERD in both groups, indicating no significant change during these blocks of MI practice in our sample. This is in contrast with several other MI practice studies showing changes in the MI-induced relative ERD with practice (Neuper et al., 2005; Stinear et al., 2006; Zich et al., 2015a, 2017a; Braun et al., 2016; Wriessnegger et al., 2018). Yet, most of these studies are NF studies, and therefore it is not possible to make general statements about enhancing effects of MI practice without NF on ERD, or to disentangle the contribution of different effects (e.g. feedback and practice effects) in this research. Further, in most of these studies much simpler movements were used, and it seems conceivable that similar to ME practice, more complex movements require more MI practice time or repetitions over several days to result in a measurable change of the MI-induced relative ERD (Jackson et al., 2003; Reiser et al., 2011). This line of argument can be seen as in contrast to the findings of significant changes in the MI event-related component N2 in a similar setup, but with even fewer trials (Allami et al., 2014). However, the adaptations of the set-up, i.e., no S1/S2 set-up and necessity to transport the device with the marble on top in a curve around the glass pane instead of transporting it in a straight line, might have increased task difficulty. Moreover, although the N2 component has been associated with motor processes, in contrast to sensorimotor ERD, research has also related it to other cognitive processes such as attention (Patel and Azzam, 2005; Folstein and Van Petten, 2008), and thus its specificity is not assured.

In the present study, Group *MI* did not improve significantly in any measure in the pre-post comparison, suggesting that MI practice including a brief familiarization phase cannot entirely replace ME when acquiring a more complex motor task. This is in line with the findings of several other studies (Mulder et al., 2004; Gentili et al., 2010; Sobierajewicz et al., 2016). During mental practice without a properly acquired internal representation of the to-be-learned movement, the state estimation (i.e., sensorimotor state, which is related e.g. to position, proprioception, velocity) may be less accurate than during ME practice (Gentili and Papaxanthis, 2015). This is presumably because ME output is compellingly combined with sensory feedback which is lacking in MI. For the present study, the lack of change in any ME-related measure for Group *MI* also argues against the possibility raised above that participants were able to form a good internal model of the movement based on Block pre-ME only, by extrapolation, or based on the video instruction. However, it is important to note that also evidence exists that does not support the suggestion that ME performance gains following MI practice without a reasonable amount of physical experience with the task. Several studies have reported improvements on behavioral and/or neural basis when only or rather almost only mentally practicing a motor task (Pascual-Leone et al., 1995; Allami et al., 2008, 2014; Ingram et al., 2019). Future research should therefore aim at further delineating the conditions under which MI practice can be an efficient replacement for physical practice.

## Conclusion

In conclusion, the present study shows a positive effect of ME practice on the subsequent execution of the movement and the ME-related ERD. In contrast to our expectations, an effect of ME practice on the MI-related ERD was not observed. Also, we could not confirm an effect of MI practice on the later execution of the movement and ME-related ERD. We conclude that both the susceptibility of MI to the beneficial effects of ME as well as the beneficial effect of MI practice on ME depend on task details. Future research should aim at delineating the relevance of task factors and thereby improve the understanding of how ME impacts on MI and vice versa.

## Data Availability Statement

The datasets generated for this study are available on request to the corresponding author.

## Ethics Statement

The studies involving human participants were reviewed and approved by the Commission for Research Impact Assessment and Ethics of the University of Oldenburg. The patients/participants provided their written informed consent to participate in this study.

## Author Contributions

CK, SD, and CZ contributed the conception of the study. MD, CK, SD, and CZ designed the study. RE supported the technical setup with contributions from MD and JW. MD and JW collected the data. MD analyzed the data and wrote the first draft of the manuscript. CK and CZ supervised MD and edited as well as commented the manuscript in all its versions. All authors contributed to manuscript revision, read, and approved the submitted version.

### Conflict of Interest

The authors declare that the research was conducted in the absence of any commercial or financial relationships that could be construed as a potential conflict of interest.

## Footnotes

^1^https://sccn.ucsd.edu/wiki/EEGLAB_Extensions
